# Oral health knowledge, attitudes and care practices of people with diabetes: a systematic review

**DOI:** 10.1186/s12889-018-5485-7

**Published:** 2018-05-02

**Authors:** Prakash Poudel, Rhonda Griffiths, Vincent W. Wong, Amit Arora, Jeff R. Flack, Chee L. Khoo, Ajesh George

**Affiliations:** 1 School of Nursing and Midwifery, Western Sydney University, Locked Bag 1797, Penrith, 2751 NSW Australia; 2 Centre for Oral Health Outcomes, Research Translation and Evaluation (COHORTE), Ingham Institute Applied Medical Research, Locked Bag 7103, Liverpool, 1871 NSW Australia; 3 Ingham Institute for Applied Medical Research, Locked Bag 7103, Liverpool, 1871 NSW Australia; 4 South Western Sydney Local Health District, Liverpool, 2170 NSW Australia; 5 South Western Sydney Clinical School, University of New South Wales, Sydney, NSW 2052 Australia; 6 School of Science and Health, Western Sydney University, Locked Bag 1797, Penrith, 2751 NSW Australia; 7 Oral Health Services and Sydney Dental Hospital, Sydney Local Health District, Surry Hills, NSW 2010 Australia; 8 Discipline of Child and Adolescent Health, Sydney Medical School, Westmead, NSW 2145 Australia; 9 School of Medicine, Western Sydney University, Locked Bag 1797, Penrith, NSW 1797 Australia; 10 Health Focus Family Practice, The Royal Australian College of General Practitioners (RACGP), National Association of Diabetes Centres (NADC), Ingleburn, NSW 2565 Australia; 11 Diabetes , Obesity and Metabolism Translational Research Unit (DOMTRU), Western Sydney University, Campbelltown, NSW 2560 Australia; 12 Faculty of Dentistry, University of Sydney, Camperdown, 2050 NSW Australia; 13 Translational Health Research Institute, Western Sydney University, Locked Bag 1797, Penrith, 2751 NSW Australia

**Keywords:** Oral health, Diabetes mellitus, Health knowledge, attitudes, practice, Review

## Abstract

**Background:**

People with uncontrolled diabetes are at greater risk for several oral health problems, particularly periodontal (gum) disease. Periodontal disease also impacts diabetes control. Good oral hygiene and regular dental visits are recommended to prevent and manage oral health problems. Several studies have been conducted to assess the oral health knowledge, attitudes, and practices of people with diabetes yet a review of these findings has not yet been undertaken. The aim of this systematic review was to synthesize current evidence on the knowledge, attitudes and practices of people with diabetes in relation to their oral health care.

**Methods:**

A systematic search of all literature was carried out in five databases using key search terms. The inclusion criteria were: 1) published in the English language; 2) from 2000 to November, 2017; 3) conducted on persons with any type of diabetes and of all ages; 4) explored at least one study outcome (knowledge or attitude or practices toward oral health care); and 5) used quantitative methods of data collection. No restrictions were placed on the quality and setting of the study.

**Results:**

A total of 28 studies met the inclusion criteria. The studies included a total of 27,894 people with diabetes and were conducted in 14 countries. The review found that people with diabetes have inadequate oral health knowledge, poor oral health attitudes, and fewer dental visits. They rarely receive oral health education and dental referrals from their care providers. Provision of oral health education by diabetes care providers and referral to dentists when required, was associated with improved oral health behaviours among patients.

**Conclusions:**

Overall, people with diabetes have limited oral health knowledge and poor oral health behaviours. It is therefore essential to educate patients about their increased risk for oral health problems, motivate them for good oral health behaviours and facilitate access to dental care.

**Electronic supplementary material:**

The online version of this article (10.1186/s12889-018-5485-7) contains supplementary material, which is available to authorized users.

## Background

In 2014, it was estimated that 422 million adults were living with diabetes mellitus (DM) worldwide [1]. The global prevalence of diabetes in the adult population has nearly doubled since 1980, rising from 4.7% to 8.5% [1]. Diabetes mellitus (DM) is a group of metabolic disorders that leads to hyperglycaemia and is classified into four general categories: type 1, type 2, gestational diabetes and other specific types of diabetes [2].

Hyperglycaemia can cause several complications related to different organ systems especially the eyes, kidneys, nerves, heart, and blood vessels [1]. Although not commonly discussed in diabetes care, people with uncontrolled diabetes are also at increased risk of developing oral health problems, particularly periodontal (gum) disease [3]. Periodontal disease, which includes both gingivitis and periodontitis, is a common inflammatory disorder caused by pathogenic microflora in the biofilm that forms adjacent to the teeth on a daily basis [4]. Gingivitis is the mildest form of periodontal disease in which inflammation is confined to the gingiva, and can be reversible with effective oral hygiene while periodontitis is the advanced stage where the inflammation extends deep into the tissues and causes loss of supporting connective tissue and alveolar bone [4]. Tissue destruction in periodontitis results in breakdown of collagen fibres of the periodontal ligament and leads to the formation of periodontal pockets between the gingiva and the tooth. Periodontitis is a slowly progressing disease but the tissue destruction is largely irreversible [4, 5]. Further, the bacteria located within the periodontal pockets are pathogenic and highly inflammatory with some having the ability to survive in the blood stream and infect other areas of the body [6, 7]. Moderate periodontitis affects approximately 40–60% of the adults worldwide [8].

It is well established that diabetes and periodontitis are directly related. Hyperglycaemia affects periodontal outcomes and periodontitis also adversely affects blood glucose levels and worsens diabetes complications. The mechanistic pathways that link diabetes and periodontitis is not clearly understood in the absence of experimental findings from clinical studies [9]. However, current information supports the potential complex interaction involving aspects of inflammation, immune functioning, neutrophil activity, and cytokine biology [9]. Hyperglycaemia is believed to enhance levels of several cytokines and mediators in saliva and gingival crevicular fluid (GCF), oxidative stress in periodontal tissues and formation of Advanced Glycation Endproducts (AGE). The AGE–RAGE (Receptor for AGE) interaction leads to the exaggerated inflammatory response and periodontal tissue destruction seen in diabetes [8]. Similarly, periodontitis promotes measures of systemic oxidative stress and raises serum levels of C-reactive protein and other acute-phase reactants and biomarkers of oxidative stress. Non-resolving chronic inflammation derived from periodontal disease also impacts on diabetes control (elevated HbA1C) and complications [8]. In light of this, current evidence from interventional studies suggests that periodontal treatment can improve blood glucose control [8, 10–12], although this evidence is often considered low quality [12] due to the heterogeneity of the studies and small sample size [13, 14].

Considering the impact of periodontal disease on diabetes and benefits of good oral health practices to minimise the risk of periodontal disease, it is important to ensure that people with diabetes are motivated to engage in good oral hygiene behaviours and are provided risk assessment and dental referrals as a part of routine diabetes care [15–17]. Several studies conducted worldwide have assessed the knowledge, attitude and practices of people with diabetes relating to oral health care, however, synthesis of these results has not yet been undertaken. Conducting such a review is important as adequate oral health knowledge or literacy is positively associated with good oral health behaviours such as, increased frequency of brushing and dental visits [18] and good periodontal health [19]. Further, oral health behaviours are influenced by the social determinants of health [20], Those who are disadvantaged or from lower socio economic groups often have unhealthy habits, poor knowledge and attitudes to oral health and uptake of dental services and therefore are more likely to suffer from the burden of oral disease [20]. Thus, the aim of this systematic review was to synthesize current evidence on the knowledge, attitudes and practices of people with diabetes in relation to their oral health care.

## Methods

This study used the PRISMA statement as a basis for reporting the systematic review findings [21, 22]. The protocol for this systematic review was not registered.

### Inclusion and exclusion criteria

All studies which met the following inclusion criteria: 1) published in the English language; 2) from 2000 to November, 2017; 3) conducted on persons with any type of diabetes and of all ages; 4) explored at least one study outcome (knowledge or attitude or practices toward oral health care); and 5) used quantitative methods of data collection, were included in this review. Intervention studies that contained baseline data on any of the study outcomes were also included. No restrictions were placed on the quality and setting of the study.

### Data sources, search strategy and study selection

A systematic literature search was carried out in the following databases: Medline, Pubmed, CINAHL, Cochrane and Embase. The keywords used in the search were: diabetes mellitus, diabetic patients, people with diabetes, oral health, dental health, oral hygiene, dental care, dental visit, knowledge, awareness, attitudes, perception, practice. Considering the database specific indexing terms, individual search strategies were used for each database. Combinations of search terms were used, including ‘Boolean’ operators (And/Or) and MeSH (Medical Subject Heading) terms. A university librarian was consulted to ensure the search strategies were appropriate and correct. The complete electronic search strategy used in Medline is presented as a supplementary file (see Additional file 1). The filter applied in the search included the language (English) and date of publication (2000–2017). A final search was carried out on 30 November 2017 to ensure the most recent literature was included in this review. In addition, the reference lists and bibliographies of all relevant studies were searched for additional sources.

**Fig. 1:**
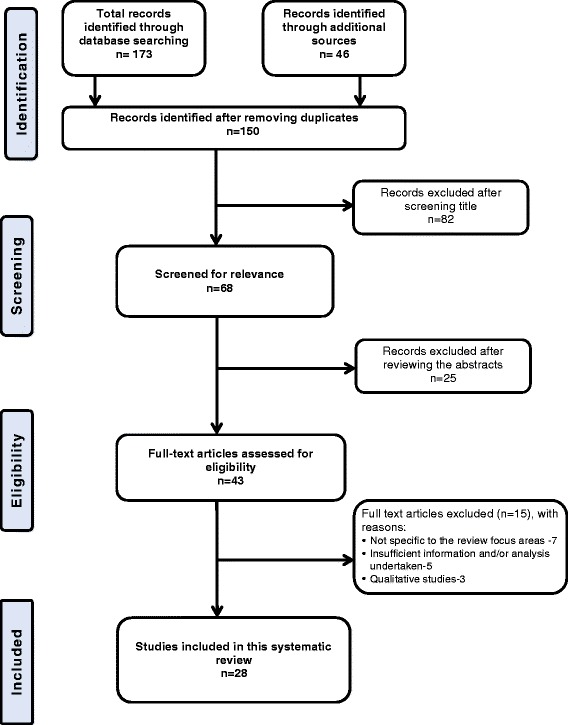
PRISMA flow chart of the study selection process

The results from the search were organised and duplicate references were removed using the Endnote bibliographic software. The title and abstract of the remaining studies were assessed by two experienced authors independently [PP (MA, MPH) & AG (MPH, PhD)] using the inclusion and exclusion criteria for suitability. In the case where it was difficult to make a decision on the basis of the title and abstract only, the full text was obtained for further assessment. Discrepancies in judgment were resolved through consultation with a third author (AA). A total of 28 studies met the inclusion criteria and were included in the review (Fig. 1).

### Quality assessment and data extraction

Two reviewers (PP & AG) independently appraised the methodological quality of the included studies using the Joanna Briggs Institute (JBI) critical appraisal checklist for analytic cross sectional studies [23] (see Additional file 2). The quality of the data was scored assigning 1 point for each applicable item with the maximum score of 8. A third author (AA) was used to reconcile any discrepancies. The scoring of each paper was calculated as percentage and the quality was rated as *** or good (with a score of 80–100%), ** or fair (50–79%), and * or low (< 50%) [24]. No articles were rejected based on quality appraisal alone.

A data extraction form was developed and piloted independently by two authors (PP & AG) and modified as required (see Additional file 3). The information including author, year of publication, country, characteristics of sample/questionnaire and key outcome items was extracted by one author (PP) and checked by two others (AA & AG) for accuracy and completeness of the results. There was clear heterogeneity among the studies in their approaches to measuring and reporting the knowledge, attitudes, and practices in relation to oral health care. Therefore descriptive analysis was carried out in most sections and data was pooled to calculate mean percentage wherever studies had similar outcome items to compare.

### Definition of terms

The term ‘people with diabetes’ has been used throughout the paper to include patients with any type of diabetes and of any ages. The ‘knowledge’ is used to include awareness and understanding of the people with diabetes relating to oral health-diabetes link, awareness about oral health complications and importance of diabetes control to minimise oral health risks. Similarly, ‘attitude’ is used to report perception and beliefs of the people with diabetes regarding oral health-diabetes relationship, oral health quality of life, and barriers in accessing oral care practices. The term ‘diabetes care providers’ refers to the diabetes healthcare team other than oral health professionals, which includes general practitioners, endocrinologists, diabetes educators, dietitians, physiotherapists and exercise physiologists.

## Results

All studies (*n* = 28) used a cross sectional design (including a intervention study) to capture the information on the knowledge, attitude and practices of patients in relation to oral health care. Of these, 4 studies used existing data or followed up participants previously included in state and national surveys [25–28]. The studies originated from 14 countries namely, United States of America (USA; *n* = 8), India (*n* = 5), Saudi Arabia (*n* = 2), Malaysia (*n* = 2), Pakistan (*n* = 2), United Kingdom (UK; *n* = 1), Sweden (*n* = 1), Ireland (*n* = 1), Finland (*n* = 1), Brazil (*n* = 1), United Arab Emirates (UAE; *n* = 1), Jordon (1) South Korea (*n* = 1) and Iran (*n* = 1). The sample size of the studies ranged from 50 to 12,405 participants with a total of 27,894 people with diabetes. Three studies surveyed the participants with type 1 DM [29–31], 6 involved participants with type 2 DM [32–37] and the remaining included both types of DM as well as people with unknown type of DM (Table 1). A variety of questionaries were used to assess oral health knowledge, attitude, and care practices of people with diabetes. Only 9 studies used a validated questionnaire or items, while remaining did not provide any clear information in this area. The questionnaires included in the studies contained items ranging from 4 to 40. Of the total studies, 4 were rated as good quality (score ≥ 80), 23 fair (score 50–79%) and the remaining as low quality (score <  50%) (see Additional file 2).

**Table 1:** Summary of the included studies with main results

Author, Year, Country	Sample/ Questionnaire characteristics	Results		Quality Rating
Yuen et al. 2009, USA [18]	253 (T1DM and T2DM); ≥18y/ 20-Q; V	K	▪ OH~DM: 47% ▪ Adequate OH knowledge significantly associated with brushing (twice/day), flossing (once/day), and dental visit (twice/year) (*P* < 0.01) ▪ Receiving OH information significantly associated with adequate OH knowledge (*P* = 0.008)	b
		P	▪ Brushing: 61.2% ▪ Flossing: 34.9%; never: 35.3% ▪ Dental visit: 58.6%	
Tomar et al. 2000, USA [25]	*N* = 4570 (DM), 101,148 (NDM); ≥25 y/ 4-Q; V	A	▪ Reasons behind not visiting dentists: perceived need to visit a dentist (37.2%), cost (28.6%), fear/anxiety (10.5%), and other reasons (23.7%)	a
		P	▪ Dental visit: PWD 65.8% vs NDM 73.1% (*P* = 0.0000); result was consistent even after controlling confounders and other correlates: sex, age, race or ethnicity, educational attainment, income, and dental insurance coverage (OR 0.82, 95% CI 0.73–0.93)	
Macek et al.,2008, USA [26]	*N* = 725 (DM), 7816 (NDM); ≥25 y	P	▪ Dental visit: 56.8% PWD vs 64.7% NDM; result remain consistent even after adjusting periodontitis status, age, sex, race/ethnicity, education, poverty status and dental insurance status	a
Moffet. 2010, USA [27]	*N* = 12,405 (DM) Q: V	P	▪ Dental visit: 77% of patients (82% with dental insurance vs 61% without dental insurance (age sex adjusted OR 2.66, 95%CI 2.33–3.0).	a
Oh et al. 2012, USA [28]	*N* = 1209 (DM) 9140 (NDM); ≥45 y	P	▪ Dental visit: 72.7% PWD vs 83.5% NDM (95% CI: 82.6%–84.4%, *p* < .0001) ▪ Diabetes status adversely affected the rate of preventive dental care ▪ Adults from racial/ethnic minority background (OR = 0.51, 95% CI: 0.33–0.79) or lower educational attainment (OR = 0.64, 95% CI: 0.47–0.88) had lower odds of having received preventive dental care	a
Orlando, et al., 2010, USA [29]	*N* = 89 (T1DM); 12–19 y/ 40-Q	K	▪ Perio~DM; 44% ▪ Health care providers advised PWD for dental check up (77%)	b
		A	▪ Care of their OH was as important as taking care of medical health: 49% ▪ Plaque or tartar build up was a problem: 33%	
		P	▪ Dental visit: 95.4%; majority (86.5%) paid through insurance	
Moore et al. 2000, USA [30]	*N* = 390 (T1DM), 203 age matched (NDM)	K	▪ OH would be better if not have diabetes: 18.2% ▪ Health care providers advised for oral hygiene and dental visit: 27.1%	b
		A	▪ PWD rated their overall oral health lower than control subjects ▪ The cost of dental care was main reason for avoiding routine visit	
		P	▪ Brushing: 72.2% PWD vs 80.2% NDM ▪ Flossing: 33% vs 30% ▪ Dental visit: 68.9% vs 75.7%	
Alves et al., 2009, Brazil [31]	*N* = 55 (T1DM), 55 age matched (NDM)	K	▪ None enrolled in an oral health educational program ▪ Informed to visit dentist by health professional: 65.5%	b
		A	▪ Reasons for avoiding dental visit: difficulty in scheduling an appointment (36.1%) and high treatment costs (27.8%)	
		P	▪ Brushing: 92.7% PWD vs 76.4% NDM ▪ Flossing: 30.9% vs 18.2% ▪ Dental visit: 63.8% vs. 48.7%	
Arunkumar et al. 2015. India [32]	*N* = 185 (T2DM)	K	▪ Perio~DM: 33% ▪ Informed about OH from physicians; none	b
Kejriwal et al.2014, India [33]	*N* = 300 (T2DM);18-65y/ 20-Q; V	K	▪ Increased risk for oral diseases: 50% ▪ Informed about OH from physicians: 10%	b
		A	▪ Preferred to see physicians for oral problem: 41%	
		P	▪ Brushing: 65% ▪ Dental visit (in 6 months): 27%	
Sandberg, et al.2001, Sweden [34]	*N* = 102 (T2DM), 102 age, gender matched (NDM); 34-77y	K	▪ OH~DM: 27%	
		A	▪ Perceived satisfaction with teeth and mouth: satisfied (83.3%), dissatisfied (16.7%) ▪ Main reason for avoiding dental visits: belief that it was not necessary	b
		P	▪ Brushing: ≥ 1times: 91.3% ▪ Dental visit: 85.1% PWD vs 95.1% NDM (*P* < 0.05)	
Lee et al. 2009, South Korea [35]	*N* = 75 (T2DM)	A	▪ 62.7% perceived their OH status as poor with 37.3% perceived as good	b
		P	▪ Brushing: 90.6% ▪ Dental visit (within 6 months): 45.3%	
Sahril et al. 2014, Malaysia [36]	*N* = 4017 (T2DM); ≥18 y	K	▪ OH~DM: 35.5%	b
		A	▪ Wanted dental referral: 59.9% ▪ Reasons not wanting a referral: perceived lack of necessity, absence of dental problems and perception that dental problems were not serious	
		P	▪ Dental visit: 16.7%; highest among: 18–19 y, lowest: **≥**70 yrs	
Aggarwal et al. 2012, India [37]	*N* = 500 (T2DM); ≥35 y/	K	▪ OH~DM: 38.4% ▪ Never received a referral for dental care: 79.4%	b
		A	▪ Avoiding dental visits due to unpleasant experience: 18.4%	
		P	▪ Brushing: 33.4% ▪ Dental visit: 75.6%; visited for regular dental checkups: 10.8%	
Al Habashneh et al. 2010, Jordon [38]	*N* = 405 (DM); RR 81% 33-Q	K	▪ Perio~DM: 47.7%; source of information: diabetes nurse (43%), physicians (38%), dentist (30%),	b
		A	▪ Did not pay attention to bleeding gums: 13.7% ▪ Rated their overall oral health as poor: 60%	
		P	▪ Brushing: 28.1% ▪ Dental visit (regular): 10%	
Allen et al., 2008, Ireland [39]	*N* = 101 (DM) 31-79y/ 20-Q; V	K	▪ Perio~DM: 33%; source of information: dentist (51%), diabetes care providers (32%)	b
		A	▪ Would choose to save a painful posterior tooth: 32%	
		P	▪ Dental visit: 42.5%; not attended for > 5 yrs.: 34%	
Badiah et al. 2012. Malaysia [40]	*N* = 102 (DM) RR 93%/ 10-Q; V	K	▪ Perio~DM: 26.5% ▪ Needs to be extra careful on oral health practices: 19.6% ▪ Those who were aware of the risk and the need for extra oral health practice were more among those who brushed at least twice a day and regular attendees	b
		P	▪ Brushing: 80.4% ▪ Dental visit (1-2y): 33.3%	
Bahammam .2015, Saudi Arabia [41]	*N* = 454 (T1DM & T2DM); RR-87%.	K	▪ Perio~DM: 46.7% ▪ Gum disease makes it harder to control diabetes: 21.8% ▪ Participants who had regular dental visits had significantly greater awareness of the Perio~DM link (P < 0.05)	b
		P	▪ Brushing: 26.8%, ▪ Flossing: occasional: 23.2%; never:73.6% ▪ Dental visit: 12.6%	
Bowyer et al. 2011, UK [42]	*N* = 229 (T1DM & T2DM); ≥ 25 y; RR 37.2%	K	▪ Aware of mouth dryness: 43% ▪ Never received any OH advice: 69.1%	b
		A	▪ Reasons for avoiding dental visit: cost (43.9%), lack of need (37.6%) and unpleasant visit (19.1%)	
		P	▪ Brushing: 67.2% ▪ Flossing: 15.3% ▪ Dental visit: 85.2%	
Kamath,net al.2015, India [43]	*N* = 137 DM RR 90.6%	K	▪ Perio~DM: 22.5%	c
		P	▪ Brushing: 33.3% ▪ Dental visit: 27.5%	
Mirza et al. 2007, Pakistan [44]	*N* = 240 (T1DM & T2DM)/ Q;V	K	▪ Aware about OH complications: 35.4% ▪ OH Knowledge was significantly related to brushing frequency (*p* = 0.005) as counselled patients brushed more frequently than uncounselled (53.4% vs 22.3%)	b
		A	▪ Denied of DM~OH: 7.6% ▪ If advised about their predisposition to oral disease, willing to increase brushing frequency (45%) and consult a dentist (23%). Nevertheless, some (31.5%) were not reluctant to change	
		P	▪ Brushing: 24%	
Sadeghi et al. 2014, Iran [45]	*N* = 200 (DM) Q; V	K	▪ OH~DM: 36.5%; source of information: dentist (65%), physicians (35%)	b
		P	▪ Brushing: 7%; no brushing: 49.5% ▪ Dental visits: 83%	
Al Amassi et al.2017, Saudi Arabia [46]	*N* = 278 (DM); 18 -64y/ 20-Q	K	▪ Perio~DM: 75.9%; source of information: media (31%), dentist (23%), physicians (21%) ▪ Controlling diabetes is important to minimize OH complications: 74.4% ▪ Patients with higher levels of education had greater awareness of the increased risk of OH problems and had better oral hygiene practices than those with lower levels of education (p < 0.05)	c
		P	▪ Brushing: 19.1% ▪ Regular dental visit: 15.1%	
Bangash et al. 2011, Pakistan [47]	300 (T1DM & T2DM)/ Q;V	K	▪ DM~OH: 64%; source of information: physicians (35%) and dentists (65%)	b
		A	▪ Denied existence of a link OH~DM: 23% ▪ Would increase brushing frequency if told of their predisposition to oral disease: 30%	
		P	▪ Brushing: 86%	
Ummadisetty et al. 2016, India [48]	*N* = 60 (DM),143 (NDM); 40-55y/ Q;V	K	▪ Perio~DM: 61.7%; source of information: physicians (36.6%) and dentist (30.69%) ▪ Physicians advised to visit a dentist: 46%	
Eldarrat. 2011, UAE [49]	*N* = 100 (DM) RR 50%	K	▪ Perio~DM: 60%	b
		A	▪ Main reason of dental visit: due to pain/discomfort	
		P	▪ Brushing: 31%; did not brush daily: 19% ▪ Flossing: once a day: 11%; never: 66% ▪ Dental visit: 40%	
Karikosk et al. 2002, Finland [50]	*N* = 336 (T2DM); 1 ≥ 18 y/ 29-Q	A	▪ Main reason for not seeing a dentist: not having any problems (95%) ▪ Important for the diabetes nurse to also offer advice about dental care: 92%	b
		P	▪ Brushing: 38% ▪ Dental visit: 63%	
Kanjirath,P.P, 2011, USA [52]	*N* = 77 (DM) and 366 (NDM)	P	▪ Brushing: 31.5% PWD vs 49% NDM ▪ Flossing: 19.4% vs 26.7.% ▪ Dental visit: 86.7% vs 82.2%	b

*K* Knowledge, *A* Attitudes, *P* Practices; Brushing ≥2times/day; Flossing≥1time/week; Dental visits: ≥1 time in the last 12 months; T1: Type 1; T2: Type 2; *DM* diabetes mellitus, *NDM* Non diabetes mellitus, *y* year, *RR* response rate, *Q* questionnaire/items, *V* validated questionnaire/items, *Perio* Periodontal disease, *OH* Oral health, *PWD* People with diabetes

^a^all or most of the criteria have been fulfilled (a score of 80–100%); ^b^some of the criteria have been fulfilled (50–79%); and ^c^few or none of the criteria have been fulfilled (< 50%) [24]

### Oral health knowledge

The majority of studies (21/28) explored the oral health knowledge of people with diabetes. The knowledge items included in the studies assessed the level of information of the patients on the risk of oral health problems in relation to diabetes, importance of good diabetic control and preventive oral health behaviours (brushing, flossing and regular dental visits) to reduce the risk for oral health problems. Majority of the studies reported that more than half of people with diabetes were unaware of the link between diabetes and oral health and their increased risk for various oral health complications including periodontal disease [18, 29–32, 34, 36–45]. In contrast, few studies did show that most participants (type 1 DM and type 2 DM) had knowledge on the link and oral health risks and this information was received mainly from dentists, physicians, and media [46–48]. Furthermore, some studies showed that those who were better informed or had good knowledge of the link between diabetes and oral health were more likely to adopt good oral health behaviours [44–47]. However, two studies which included matched controls found that individuals with diabetes had lower oral health knowledge than those without diabetes [30, 31].

A survey conducted in the USA concluded that adequate oral health knowledge had a statistically significant relationship with the frequency of brushing (at least two times daily), flossing (at least once a day) and dental visits (at least two times a year) (*p* = < 0.01) [18]. Similarly, adequate oral health knowledge was also significantly associated with other factors such as, higher level of education (*p* = 0.05) [41] and having received oral health information (*p* = 0.008) [18, 46]. Studies reported that the majority of the patients did not receive any oral health information from general physicians or diabetes care providers [29–33, 42, 46, 49]. However, few studies such as, those conducted in USA [29] and Brazil [31] indicated that majority (77 and 65.5% respectively) of patients were advised by health professionals for dental checkups [29].

### Oral health attitudes

The attitudes of people with diabetes towards oral health were reported in 15 studies. The relevant attitude items related to perceived need and importance of oral health, self-rating of oral health status, agreement/disagreement on the link between diabetes and oral health, and reasons for refusing dental referrals/visits. Studies reported that the perceived need [42] and importance [39] of oral health care in relation to diabetes was poor among people with diabetes [39, 42]. Some studies revealed that patients with diabetes rated their overall oral health status as poor [35, 38] and this was lower compared to those without diabetes [30]. Comparison between nations revealed that participants from high income nations perceived their oral health status higher [30, 34] than those from low income nations [35, 38]. A study conducted in the USA showed that about half of the participants (49%) acknowledged that taking care of their oral health was as important as their general health, and only a third (33%) considered plaque or tartar build up as a problem [29]. Furthermore, some participants also denied that there was a link between diabetes and oral health [44, 47].

A survey conducted in Malaysia revealed that half (51%) of the people with diabetes believed teeth problems were not serious and this belief was one of the main reasons behind refusing a dental referral [36]. A number of reasons were highlighted by participants for not having regular dental visits, the most notable being the cost of dental care, lack of need for oral health care, absence of dental problems, unpleasant dental visits and difficulty in scheduling an appointment [30, 31, 36, 37, 42, 50]. The cost of dental care was the main underlying reason behind lower dental visits in studies from high income countries [30, 42] while the perceived lack of necessity, discomfort and fear of oral health care were the main reasons for the participants from low income countries [36, 37]. Generally, participants from low income countries had a tendency to see the dentist for urgent treatments only [37, 49]. Similarly, a study conducted in Ireland reported that 32% of the participants would choose to save a painful posterior tooth [39].

The low perceived need for dental care among participants was also attributed to their lack of oral health knowledge and information [44]. Nearly half of the participants (45%) from a study conducted in Pakistan stated that they would engage in more positive oral health practices if they were informed about the risks and consequences of poor oral health [44]. A study from Finland showed that almost all of participants (95%) were receptive to receive advice on oral health [50] from diabetes care providers. However, less than one third of participants (31%) from another study also stated that any oral health information provided would not affect their oral hygiene behaviours and dental checkups routines [44]. Similarly, some participants (41%) in a study conducted in India also preferred to consult physicians for oral problems [33].

### Oral health care practices

Oral health care practices were reported in most of the studies (*n* = 25) and addressed the patients’ frequency of brushing, flossing, and dental visits. In the studies (*n* = 18) that reported frequency of brushing, just less than half of the participants who have diabetes (mean 49.3%, 95% CI 35.70–62.90) brushed twice a day [24, 27–29, 31, 33–37, 39–42, 44, 45, 48]. Four studies presented data on regular flossing (≥1/day) by patients and only a quarter of them (mean 25.1%, 95% CI 10.36–39.88) undertook flossing at least once a day. Overwhelmingly, regular dental visits among the people with diabetes were also lower. Across 20 studies just over half of the people with diabetes (mean 54%, 95% CI 42.80–65.25) had dental visits in the last 12 months [24–29, 31, 33–37, 39–45, 48]. In addition, the uptake of dental services was very low (mean 34.6%, range 10%–75.60%) in low or middle income countries [51], such as, India [33, 37, 43], Malaysia [36, 40] and, Jordan [38] compared with high income countries [51] (mean 60.6%, range 12.6%–95.4%), which included USA [18, 25–27, 29, 52], UK [42], Finland [50], Sweden [34], Ireland [39], UAE [49], Saudi Arabia [41, 46] and Korea Republic [35]. Within the high income countries lower rate of dental visits was observed in Asian countries (range 12.6%–45.3%) [49] [41, 46] [35] which was similar to other low income countries (10%–45%), except the one study from India which reported a dental visit rate of 75.6% [37]. However, a study conducted in Ireland also showed a lower compliance of dental visits with only 43% of the participants visiting a dentist in the last year and 34% reported not attending a dentist for more than 5 years [25]. More than one third (37%) of patients with diabetes included in a Finnish study did not visit a dentist despite being entitled for state-subsidized dental care [50].

Furthermore, compared with age matched controls of subjects without diabetes, people with diabetes had a lower dental visit frequency (68.9% vs 75.7%) [30]. This result is consistent with another study involving a national sample (*n* = 4570) which also found that the people with diabetes were less likely to visit a dentist than those without diabetes (65.8 vs 73.1%, *P* = 0.0000) [25]. The pattern of visits remained unchanged even after controlling for confounders such as age, race or ethnicity, educational level, income level and dental insurance coverage (OR 0.82, 95% CI 0.73–0.93) [25]. A similar result was obtained from another study conducted in the USA, which used data from a national survey (56.8% Vs 64.7%, OR 0.72, 95%CI 0.53–0.96) [26]. In contrast, a study conducted in Brazil involving children with type 1 diabetes, reported that frequency of dental visits was found to be higher compared to matched control non diabetic children (63.8% vs 48.7%,) [29].

Receiving oral health information was found to have a significant impact in improving good oral health care practices among participants [18]. Studies found that participants who were advised by health professionals to have regular dental checkups and instructed on tooth brushing and flossing were more likely to visit a dentist once in a year (*P* = 0.002) and to brush and floss teeth at least twice daily (*P* = 0.006) [18]. Similar results were found in the study from Pakistan where the knowledge about oral complications provided by physicians was significantly associated with brushing frequency (*P* = 0.005), where 53.4% of counselled patients brushed twice or more daily compared to 22.3% patients who were not counselled [44].

## Discussion

The focus of this review was to provide a synthesis of current evidence on knowledge, attitudes and practices of people with diabetes in relation to their oral health care. The questionnaire and methods used to conduct surveys in this area were largely varied and hence the reliability of the studies included in this review may be compromised. More than half of the studies also failed to provide information about the validity of the tools used to measure knowledge, attitude and practices. Furthermore, almost all of the studies used convenience sampling and most did not report the response rate or any comparison between the respondents and non-respondents. Of the total studies included in this review only four were rated as of good quality.

Overall, the results of this review show that a majority of people with diabetes are unaware of the bidirectional link between diabetes and periodontal disease and they have limited knowledge of their risks for oral health problems [18, 29–32, 34, 36–45]. As could be expected, knowledge of oral health risks was found to be significantly associated with better oral health care and practices [18, 44]. However, a majority of people with diabetes did not receive information on oral health risks in relation to their diabetes or advice on oral health care from diabetes care providers [29–33, 42, 46, 49]. This finding is similar to the results from a recent scoping review which explored the knowledge and practices of diabetes care providers in oral health care and found that they generally do not provide any information on oral health care to their patients in the diabetes care settings [17]. According to the review the main barriers facing diabetes care providers in this area include inadequate knowledge about the oral health-diabetes bidirectional relationship, unavailability of oral health assessment tools/guidelines and referral pathways for promoting oral health [17].

In spite of being at high risk for developing oral health problems, the perceived need and importance for oral health care among people with diabetes is found to be very poor [39, 42], as it appears oral health is not as important as general health for these patients [29, 36]. People with diabetes rated their overall oral health lower [35, 38] than that those without diabetes [30]. The cost of dental care, lack of dental care need, unpleasant dental visits, and difficulty in scheduling appointments were found to discourage people from seeking dental care [30, 31, 36, 37, 42, 50]. Despite these barriers, this review also found that people with diabetes are more likely to engage in positive health behaviours if they are informed about the risks and consequences of poor oral health. Patients were also found to be interested in receiving oral health information from diabetes care providers [50].

Most notably, people with diabetes were found to have poor compliance with oral hygiene behaviours and dental visits as less than half of the patients (49.3%) reported brushing twice a day. In addition, flossing of teeth appeared to be least important for patients with diabetes with only a quarter (25.1%) of the participants reported flossing their teeth everyday to clean interdental surfaces. Similarly, just over half (54%) of the people visited a dentist in the 12 months. Large national studies have also reported a lower frequency of dental visits among people with diabetes compared to those without diabetes [43, 49]. These figures are lower than the general population of some high income countries such as in England where a higher proportion of adults (aged 15 and over) brushed twice a day (75%) and visited dentist (73%) in the last 12 months [53]. Similarly, nearly two third of American (64% aged 18–64) [54] and Australian (60.3% aged 15 and over) [55] visited dentist in the past year [54]. This delay to seek dental care among people with diabetes is a significant concern considering periodontal disease can negatively impact on diabetes control and worsen diabetes complications [8].

This review has also identified various factors that could contribute to the poor oral health knowledge, attitudes, and behaviours among people with diabetes. One of the key factors is the limited oral health education and motivation being provided to these patients during diabetes care. It is apparent that oral health education can improve knowledge, attitudes, and practices regarding oral health [56] and therefore it is very important to include it in diabetes patient education, which is a vital and integral component of successful diabetes care [57]. Such education should include aspects of good oral hygiene practices as these are important to prevent gingivitis (earlier stage of gum disease) and control of advanced periodontal lesions [58].

Another major barrier identified in this review is the cost of dental care, which is often cited as an issue among people with chronic conditions [59]. Treatment of oral health problems is often costly and has been a significant economic burden for many high income countries [60]. Similarly, accessible dental care services is also important considering the fact that a study from Sweden reported more than one third of people with diabetes did not visit the dentist despite being entitled for subsidised dental care. Similar results are also found in the general population in Australia where a national oral health survey reported that there were no significant differences in dental visit between adults eligible for public dental services and those who were not eligible (both 62%) [55]. Although it is not specified in the study from Sweden, it is possible that the lengthy waiting time to access in public/subsidised dental care services [59, 61] may deter people with diabetes from visiting a dentist. Therefore, the feasibility of setting up affordable and accessible dental referral pathways for people with diabetes also needs to be explored as such preventative initiatives could ultimately be more cost effective [62] than delaying dental treatment until severe oral complications have developed. Most importantly, the identification and treatment of periodontal disease is particularly relevant for this at risk population as it could potentially improve their diabetes control [11–16]. However, strategies to improve the oral health of people with diabetes may need to be tailored to high and low income countries particularly since oral health is not a priority for patients in low income countries and dental problems are often left untreated [63]. Furthermore, there is insufficient emphasis on primary prevention of oral diseases and limited access to oral health care [63] in developing countries. In this context, population based preventive oral health programs could be more effective.

It is also important to consider that while diabetes care involves a multidisciplinary team including general practitioners, endocrinologists, diabetes educators, dietitians, podiatrist and physiotherapists, dentists are usually not included as part of this care team, despite the impact of poor oral health on diabetes control. Considering the findings of this review, both diabetes care providers and dentists have an excellent opportunity to collaborate and increase awareness among patients with diabetes of their increased risk of oral health problems and motivate them to have good oral hygiene behaviours and regular dental visits. The involvement of dentists in multidisciplinary teams has shown to have a positive impact in other clinical areas like antenatal care. For example, the Midwifery Initiated Oral Health (MIOH) program in Australia where dentists and midwives work in partnership has demonstrated a significant improvement in the oral health knowledge and confidence of midwives to promote oral health as well as the oral health knowledge, quality of life, uptake of dental services and oral health status of pregnant women [64, 65].

### Implication of the findings

The results of this review have several implications for diabetes care providers, oral health professionals and policy makers. Diabetes care providers should play a more active role in promoting oral health among their patients. They should educate patients about their increased risk for oral health complications and advise them to have regular dental checkups. Diabetes care providers may also need to improve their own knowledge in this area in order to incorporate oral health promotion into their practice. Oral health professionals should inform people with diabetes about good oral health behaviours and emphasize the importance of good diabetes control in minimising oral health risks. In addition, policy makers need to develop and implement standardised oral health care guidelines and oral health promotional resources for diabetes care settings as well as create appropriate referral pathways to increase uptake of dental services for this at risk population.

### Limitations

The studies in the review vary in quality and have several common methodological limitations. These include: lack of reported response rates, varying questionnaires used to measure study outcomes; limited validated questionnaires and inadequate discussion of confounding factors that may have affected the findings (age, education, income level). Studies included were from both high and low income countries and therefore it is not known whether the different health care systems and cultural beliefs across these countries could have affected the knowledge, attitudes and practices of people with diabetes in relation to oral health care. Self-reported data from the studies also limit the generalisation of the findings. The systematic review undertaken also has limitations. The review did not look for unpublished articles as well as those published in other languages and hence there is a possibility that we may not have retrieved all studies in this area. There is also the possibility of outcome reporting bias. Future studies in this area need to be designed taking these limitations into consideration to ensure high quality evidence that is reproducible and generalizable.

## Conclusions

This systematic review confirms that people with diabetes have inadequate oral health knowledge, poor oral health attitude, and lower compliance of recommended oral hygiene behaviours and dental visits. They are also not receiving adequate oral health information and care advice from diabetes care providers. It is important that people with diabetes are educated about their increased risk of oral health complications and encouraged to seek regular dental checkups. A multidisciplinary approach involving oral health professionals is needed to capacity build diabetes care providers to promote oral health and encourage their patients to seek dental care along with the establishment of appropriate and affordable dental referral pathways.

## Additional files

Additional file 1: Full search strategy in Medline. (DOCX 13 kb)

Additional file 2: Appraisal of methodological quality of the studies. (DOCX 33 kb)

Additional file 3: Data Extraction Form. (DOCX 17 kb)
