# Supplementary material for: Oral health knowledge, attitudes and care practices of people with diabetes: a systematic review
Source: BMC Public Health. 2018 May 2;18:577. doi: 10.1186/s12889-018-5485-7 (PMC5930945; doi:10.1186/s12889-018-5485-7)
Supplement: Supplementary file 1 — Full search strategy in Medline. (DOCX 13 kb) [file 12889_2018_5485_MOESM1_ESM.docx]

**Additional file 1: Full search strategy in Medline**

1. exp Diabetes Mellitus, Experimental/ or Pregnancy in Diabetics/ or exp Diabetes Mellitus, Type 1/ or Diabetes Mellitus/ or exp Diabetes Mellitus, Type 2/

2. "people with diabetes".mp.

3. "patient with diabetes".mp.

4. "diabetic patients".mp.

5. diabetics.mp.

6. 1 or 2 or 3 or 4 or 5

7. exp Knowledge/ or exp Health Knowledge, Attitudes, Practice/

8. *Awareness/ or awareness.mp.

9. understanding.mp.

10. *Perception/

11. *Toothbrushing/

12. exp Oral Hygiene/ or flossing.mp.

13. *Oral Health/ or *Oral Hygiene/ or *Dental Care/ or dental health.mp.

14. dental visit.mp.

15. "dental checkup".mp.

16. 7 or 8 or 9 or 10

17. 11 or 12 or 13 or 14 or 15

18. 6 and 16 and 17

19. limit 18 to (english language and yr="2000 - 2017")
