# Supplementary material for: Oral health knowledge, attitudes and care practices of people with diabetes: a systematic review
Source: BMC Public Health. 2018 May 2;18:577. doi: 10.1186/s12889-018-5485-7 (PMC5930945; doi:10.1186/s12889-018-5485-7)
Supplement: Supplementary file 2 — Appraisal of methodological quality of the studies. (DOCX 33 kb) [file 12889_2018_5485_MOESM2_ESM.docx]

**Additional file 2: Appraisal of methodological quality of the studies**

|  | Methodological items | | | | | | | | Score | % |
| --- | --- | --- | --- | --- | --- | --- | --- | --- | --- | --- |
|  | 1 | 2 | 3 | 4 | 5 | 6 | 7 | 8 |  |  |
| Study reference | Inclusion  Criteria defined? | Subjects and setting described in detail? | Exposure measured reliable/valid? | Measurement of condition  Standard? | Confounding factors identified? | Confounding factors addressed? | Outcomes  Measured in a valid way? | Statistical analyses appropriate? |  |  |
| Yuen et al. 2009, USA[18] | + | + | N/A | N/A | - | - | + | + | 4/6 | 67% |
| Tomar et al. 2000, United States of America (USA) [25] | + | + | N/A | N/A | + | + | + | + | 6/6 | 100% |
| Macek et al.,2008, USA [26] | + | + | N/A | N/A | + | + | - | + | 5/6 | 83% |
| Moffet. 2010, USA [27] | + | + | N/A | N/A | + | + | + | + | 6/6 | 100% |
| Oh et al. 2012, USA[28] | + | + | N/A | N/A | + | + | - | + | 5/6 | 83% |
| Orlando, et al, 2010, USA[29] | + | + | N/A | N/A | - | - | - | + | 4/6 | 67% |
| Moore et al. 2000, USA[30] | + | + | N/A | N/A | - | - | - | + | 3/6 | 50% |
| Alves et al. 2009, Brazil[31] | + | + | N/A | N/A | - | - | - | + | 3/6 | 50% |
| Arunkumar et al. 2015. India[32] | + | + | N/A | N/A | - | - | - | + | 3/6 | 50% |
| Kejriwal et al.2014, India[33] | + | + | N/A | N/A | - | - | + | + | 4/6 | 67% |
| Sandberg, et al.2001, Sweden[34] | + | + | N/A | N/A | - | - | - | + | 3/6 | 50% |
| Lee et al. 2009. South Korea[35] | + | + | N/A | N/A | + | - | - | + | 4/6 | 67% |
| Sahril et al. 2014, Malaysia[36] | + | + | N/A | N/A | - | - | - | + | 3/6 | 50% |
| Aggarwal et al. 2012, India[37] | + | + | N/A | N/A | - | - | - | + | 3/6 | 50% |
| Al Habashneh et al. 2010, Jordon[38] | + | + | N/A | N/A | - | - | - | + | 3/6 | 50% |
| Allen et al., 2008, Ireland[39] | + | + | N/A | N/A | - | - | - | + | 3/6 | 67% |
| Badiah et al. 2012, Malaysia[40] | + | + | N/A | N/A | - | - | + | + | 4/6 | 67% |
| Bahammam.2015, Saudi Arabia[41] | + | + | N/A | N/A | - | - | - | + | 3/6 | 50% |
| Bowyer et al. 2011, United Kingdom[42] | + | + | N/A | N/A | - | - | - | + | 3/6 | 60% |
| Kamath et al. 2015, India[43] | _ | + | N/A | N/A | - | - | - | + | 2/6 | 33% |
| Mirza et al. 2007, Pakistan[44] | + | + | N/A | N/A | - | - | + | + | 4/6 | 67% |
| Sadeghi et al. 2014, Iran[45] | + | + | N/A | N/A | - | - | + | + | 4/6 | 67% |
| Al Amassi, et al.2017, Saudi Arabia[46] | + | + | N/A | N/A | - | - | - | + | 3/6 | 50% |
| Bangash et al. 2011, Paikistan[47] | + | + | N/A | N/A | - | - | + | + | 4/6 | 67% |
| Ummadisetty et al. 2016, India[48] | + | + | N/A | N/A | - | - | + | + | 4/6 | 67% |
| Eldarrat. 2011, United Arab Emirates[49] | + | + | N/A | N/A | - | - | - | + | 3/6 | 50% |
| Karikosk et al. 2002, Finland[50] | + | + | N/A | N/A | - | - | - | + | 3/6 | 50% |
| Kanjirath, 2011, USA[52] | + | + | N/A | N/A | - | - | - | + | 3/6 | 50% |

+ satisfies criteria, - does not satisfy criteria
