# Supplementary material for: Oral health knowledge, attitudes and care practices of people with diabetes: a systematic review
Source: BMC Public Health. 2018 May 2;18:577. doi: 10.1186/s12889-018-5485-7 (PMC5930945; doi:10.1186/s12889-018-5485-7)
Supplement: Supplementary file 3 — Data Extraction Form. (DOCX 17 kb) [file 12889_2018_5485_MOESM3_ESM.docx]

| **Author** |  | | | | |
| --- | --- | --- | --- | --- | --- |
| **Year** |  | | | | |
| **Settings and Country** |  | | | | |
| **Title** |  | | | | |
| **Sample Size**  **Method** | **N…………….** | | | **If control (N)…………………….** | **Other comments/analysis** |
|  |  | | |  |  |
| **Type of DM** | **DM1……….** | | **DM2………….** | **Other/Unsure…………** |  |
| **Response Rate** |  | | | |  |
| **Questionnaire**  **Detail** | **Item, validation process etc.** | | | |  |
| **Study Outcomes** | **Knowledge, Attitude and Practices in relating to Oral Health** | | | |  |
| **1.Knowledge** | ☐✓  ☐✗ | 1) Aware on Periodontal/OH- diabetes link=………%  2)Aware on any OH risk=…………………….%  3) Source of information on OH Knowledge:  4) DCP/Physicians advised on:  a) Oral Hygiene……………………………  b) Dental visit ……………………………..  5) Other key findings …………………… | | |  |
| **2. Attitudes** | ☐✓  ☐✗ | 1) Perception on the link:……………....................  2) Perceived importance of OH………………….  3) Perceived OH quality ……………………………  4) Other key findings………………………………… | | |  |
| **3. Practices** | ☐✓  ☐✗ | 1) Brushing frequency (≥2times/day)=…………%  2) Flossing: (≥1time/day):……………..%  3) Dental visit rate (≥1time/yr):……………..%  4) Not attended (≥2yr): ……………..% | | |  |
| **DM: Diabetes mellitus; OH: oral health** | | | | | |

**Additional file 3: Data Extraction Form**
